# Risk Assessment of Patients Undergoing Transfemoral Aortic Valve Implantation upon Admission for Post-Interventional Intensive Care and Surveillance: Implications on Short- and Midterm Outcomes

**DOI:** 10.1371/journal.pone.0167072

**Published:** 2016-11-23

**Authors:** Fadi Al-Rashid, Philipp Kahlert, Friederike Selge, Heike Hildebrandt, Polycarpos-Christos Patsalis, Matthias Totzeck, Petra Mummel, Tienush Rassaf, Rolf Alexander Jánosi

**Affiliations:** 1 Department of Cardiology of the West-German Heart and Vascular Center Essen, Essen University Hospital, University Duisburg-Essen, Essen, Germany; 2 Department of Neurology, Essen University Hospital, University Duisburg-Essen, Essen, Germany; Azienda Ospedaliero Universitaria Careggi, ITALY

## Abstract

**Background:**

Several studies have found that standard risk scores inaccurately reflect risk in TAVI cohorts. The assessment of mortality risk upon post-interventional ICU admission is important to optimizing clinical management. This study sought to determine outcomes and factors affecting mortality in patients admitted to the intensive care unit (ICU) after transcatheter aortic valve implantation (TAVI), and to analyze and compare the predictive values of SAPS II and EuroSCORE.

**Methods and Findings:**

214 consecutive patients treated with transfemoral TAVI (2006–2012) admitted to the ICU in an academic tertiary-care university hospital, were included in this retrospective data analysis. The overall 30-day mortality rate was 7%. Non-survivors at 30-days and survivors showed differences in the rates of catecholamine therapy upon ICU admission (93 vs. 29%; *p*<0.001), stroke (20 vs. 1%;*p*<0.001), sepsis (27 vs. 2%;*p*<0.001), kidney injury (83 vs. 56%; log-rank *p*<0.001), catecholamine therapy (88 vs. 61%;log-rank *p*<0.001) and vascular complications (60 vs. 17%; p<0.001). Mean SAPS II score and predicted mortality were higher in non-survivors (38.1±7.0 vs. 29.9±6.2;p<0.001 and 23.1±11.7 vs. 10.5±8.2;p<0.001, retrospectively), whereas the logistic EuroSCORE could not discriminate between the groups (*p* = 0.555). Among the biochemical parameters, the maximum values of creatinine, procalcitonin, and troponin I during the first 48 h after ICU admission were significantly higher in non-survivors. Multivariate analysis of baseline characteristics and complications associated with two-year mortality showed no significant results.

**Conclusions:**

The SAPS II is a good tool for estimating ICU mortality immediately after performing the TAVI procedure and provides valuable information for other known predictors of mortality.

## Introduction

Transcatheter aortic valve implantation (TAVI) has become an established treatment option for inoperable patients with severe symptomatic aortic valve stenosis and is a valuable option for high-risk patients [1,2]. Approximately 30% of all aortic valve procedures in Germany are currently performed via this approach [3]. However, despite recent advances, TAVI continues to be associated with a non-negligible risk of complications such as stroke, acute kidney injury (AKI), vascular complications, or cardiac arrhythmias [4,5].

The postoperative treatment of the TAVI patients, who are usually old and present with various comorbidities, presents a new challenge for intensive care specialists. Although numerous studies have addressed perioperative anaesthetic management [6], little is known about the risk factors that lead to a longer intensive care unit (ICU) stay, which is often associated with higher mortality [7,8]. Several factors, such as the need for invasive respiratory support or catecholamine administration, are generally associated with a poorer survival rate during an ICU stay [9,10]. The impact of these factors on mid-term outcomes in TAVI patients has previously not been evaluated.

Estimation perioperative risk and mortality significantly influences the therapeutic strategy used in patients with symptomatic severe aortic valve stenosis. Several studies have found that standard risk scores (e.g., the logistic European System for Cardiac Operative Risk Evaluation (EuroSCORE), inaccurately reflect the risk in TAVI cohorts [11,12]. The assessment of mortality risk greatly influences post-interventional admission to the ICU in order to provide an optimized clinical management. The Simplified Acute Physiology Score (SAPS) II is one of the most common scores used to predict mortality and to discriminate between different patient populations [13,14] in the ICU. SAPS II has not been used for the assessment of mortality in TAVI patients admitted to the ICU for post-interventional surveillance and care, while the predictive impact of SAPS II in comparison to the EuroSCORE in this patient population is not yet known.

## Materials and Methods

### Patient Population

We retrospectively analysed a consecutive cohort of 214 inoperable and high-risk patients with severe symptomatic aortic stenosis, who underwent transfemoral TAVI and were monitored post-procedurally in our interdisciplinary medical 22-bed ICU between January 2006 and March 2012. Patients who died during the procedure or were monitored post-procedurally on one of the other five ICUs in our hospital were excluded from the analysis (due to limited availability of complete datasets). The local ethics committee of the University of Duisburg-Essen approved this retrospective analysis (No. 15-6311-BO). Patient records were de-identified and analysed anonymously.

Patients with symptomatic severe aortic valve stenosis were considered for TAVI if they had a logistic EuroSCORE ≥ 20% or surgery was considered to involve excessive risk due to comorbidities and other risk factors not reflected by the logistic EuroSCORE (e.g., frailty, porcelain aorta, or prior chest radiation). The indication for TAVI in the individual patient was decided upon by consensus of the multidisciplinary Heart Team, which included cardiologists, cardiac surgeons, and physicians from other disciples whenever needed, according to current guidelines [2].

Patients were excluded from transfemoral TAVI if the following conditions were observed: unsuitable anatomy, unprotected left main coronary disease, recent myocardial infarction or cerebrovascular event, sepsis or active endocarditis, left ventricular or atrial thrombus, active peptic ulcer, or bleeding diathesis.

For our analysis, we divided our cohort into two groups: non-survivors, consisting of all patients who died within 30 days after the TAVI procedure, and the survivors.

### TAVI Procedure

TAVI was performed by a multidisciplinary heart-team in a hybrid operating room using standard techniques [15,16], predominantly under analgosedation [17], with percutaneous femoral artery access and closure [18]. One of two currently CE-approved bioprosthesis (Edwards Sapien [n = 155] and Medtronic CoreValve [n = 59]) was implanted.

All patients were periprocedurally monitored with a six-electrode virtual 12-lead electrocardiogram and pulse oximetry. Additionally, an indwelling urinary catheter was inserted. A radial artery catheter and a triple lumen central venous catheter in the internal jugular vein (under ultrasound guidance) were placed, along with a pulmonary artery balloon catheter and a provisional pacemaker catheter [17].

### Intensive Care Unit

Study data were obtained from patients who were admitted to the interdisciplinary 22-bed ICU, which covers the neurology clinic and five specialized departments for internal medicine. In this ICU, 12 beds are devoted to internal medicine patients. The nurse-to-patient ratio was 1:3, and the ICU medical team included six doctors (two intensive care specialists, and four fellows in training), who worked in 8–12 h shifts as critical care physicians.

### Intensive Care Management

All patients were routinely transferred to the ICU after the procedure for post-interventional surveillance and further care for a minimum of 24 hours. Their vital parameters were monitored continuously and special attention was paid to rhythm disturbances, neurological disorders, access site complications, systemic blood pressure, and fluid balance.

The SAPS II and the corresponding predicted mortality rates were calculated based on data collected within the first hour after ICU admission, respectively, as described previously [13,19].

The assessment of cardiac symptoms and mental status required careful observation of the patients for the early detection of signs of acute heart failure or neurological deficits. This assessment was performed three times a day. Vascular access sites and peripheral pulses were monitored for hematoma, pseudoaneurysm, or acute thrombosis (three times a day). To prevent infections, stringent cleaning of vascular access sites and care of the urinary catheter were performed daily. Whenever signs of systemic infection were present, blood and/or urinary cultures were taken and an appropriate antibiotic therapy was initiated.

Laboratory tests were performed upon admission and the subsequent morning, if not indicated otherwise due to complications. These tests included routine blood count for detecting signs of occult bleeding, infection, dual antiplatelet therapy intolerance, or heparin-induced thrombocytopenia, and for the assessment of renal function, which was used to diagnose AKI [20]. AKI was defined as an absolute increase in serum creatinine level of ≥0.3 mg/dL (≥26.4 μmol/L), a percentage increase in serum creatinine level of ≥50%, or a reduction in urine output, defined as <0.5 mL/kg/h for more than 6 h within 48 h after TAVI, in accordance with updated VARC criteria [20].

Standard medical therapy included daily unfractionated heparin until mobilization, acetylsalicylic acid (100 mg), and clopidogrel (75 mg) daily. In cases where oral anticoagulation was required (e.g., for atrial fibrillation), patients were treated with clopidogrel and (oral) anticoagulation with substitution of clopidogrel with acetylsalicylic acid after six months to avoid triple anticoagulation therapy.

### Statistical Analysis

Data are presented as the mean ± standard deviation if normally distributed or as median and interquartile range for non-normally distributed data. Categorical variables are given as frequencies and percentages. Categorical data were compared between groups using χ^2^- or Fisher’s exact test. Continuous variables were compared using the Student t-test for dependent and independent samples or the Mann–Whitney U and Wilcoxon signed-rank tests. Survival curves were constructed using Kaplan–Meier estimates based on all available data and were compared using a log-rank test. Statistical significance was assumed when the *p*-value <0.05. Each tool’s discriminatory power was assessed by calculating the area under the receiver operating characteristic curve (AUROC). The cohort was also divided into four quartiles using the maximum value of specific lab-values for further comparison. Additionally, we performed a multivariate analysis of baseline characteristics and procedural results for two-year mortality. For this purpose we utilized a Cox regression model with forward stepwise analysis. Variables related to two-year mortality with a significance level of P < 0.1 were used. For this study, these variables included ejection fraction, mean transaortic pressure gradient, pulmonary hypertension, SAPS II, SAPS II predicted mortality, ICU stay, stroke, sepsis, catecholamine therapy, acute kidney injury, cardiopulmonary resuscitation, and maximal levels of creatinine, procalcitonin (PCT), as well as Troponin I.

All analyses were performed using PASW [SPSS] (Version 21.0, IBM SPSS, Chicago, IL, USA). The authors had full access to the data and take responsibility for their integrity. All authors have read and agreed to the manuscript as written.

## Results

### Patient characteristics

Our study cohort represents a typical transfemoral TAVI population with severe, symptomatic aortic stenosis and high operative risk due to age and comorbidities (Table 1). Compared with 30-day survivors, non-survivors had a lower ejection fraction than did survivors (39 ± 15% vs. 48 ± 13%; p = 0.008). Notably, preinterventional risk score assessment by the logistic EuroSCORE failed to discriminate between survivors and non-survivors, both at 30 days and two years (see below).

**Table 1:** Patient Characteristics.

	All patients n = 214	Survivor n = 199	Non-Survivor n = 15	p-value
Age [yrs.], mean±SD	80 ± 6	80 ± 6	81 ± 4	0.519
Male sex, n (%)	88 (41)	80 (40)	8 (53)	0.455
Logistic EuroSCORE [%], mean±SD	20 ± 12	20 ± 12	22 ± 16	0.555
SAPS II predicted mortality, mean±SD	11.4 ± 9.1	10.5 ± 8.2	23.1 ± 11.7	<0.001
Arterial hypertension, n(%)	204 (95)	190 (95)	14 (93)	0.124
Hyperlipidaemia, n(%)	163 (76)	154 (77)	15 (100)	0.052
Diabetes mellitus, n(%)	74 (35)	70 (35)	4 (27)	0.405
Obesity, n(%)	84 (39)	79 (40)	5 (33)	0.498
Coronary artery disease, n(%)	155 (72)	146 (73)	9 (60)	0.133
Myocardial infarction <90 days, n(%)	8 (4)	8 (4)	0 (0)	0.415
Previous percutaneous coronary intervention, n(%)	80 (37)	75 (38)	5 (33)	0.600
Prior cardiac surgery, n(%)	31 (15)	30 (15)	1 (7)	0.333
Ejection fraction [%], mean±SD	48 ± 13	48 ± 13	39 ± 15	0.008
Chronic obstructive lung disease, n(%)	51 (24)	47 (24)	4 (27)	0.910
Estimated glomerular filtration rate, mean±SD	50 ± 19	51 ± 19	47 ± 19	0.425
Creatinine [mg/dl], mean±SD	1.6 ± 1.1	1.6 ± 1.1	1.8 ± 1.2	0.513
Pulmonary hypertension, n(%)	25 (12)	23 (12)	2 (13)	0.916
Peripheral artery disease, n(%)	40 (19)	33 (17)	7 (47)	0.007
NYHA functional class, median(range)	3 (2–4)	3 (2–4)	3 (2–4)	0.356
Aortic valve area [cm^2^], mean±SD	0.6 ± 0.2	0.6 ± 0.2	0.6 ± 0.1	0.588
Mean transaortic pressure gradient [mmHg], mean±SD	45 ± 18	46 ± 18	37 ± 18	0.072

**SAPS**: Simplified Acute Physiology Score, **ICU**: Intensive care unit, **NYHA**: New York Heart Association classification.

### Mortality

The overall 30-day and two-year mortality rates were 7.5% and 22.4%, respectively. The mortality rate of the previously defined “non-survivors” group, which consisted of all patients who died within 30 days after the TAVI procedure, was 7% (n = 15). Nearly all of these patients (14 of 15; 93%) died during their in-hospital/ICU stay.

**Fig 1:**
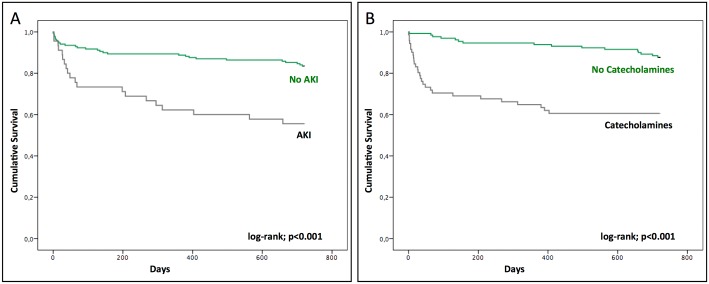
Survival. Survival rate, as indicated by the rate of peri-procedural acute kidney injury (A) and to the need for catecholamine adminstration (B).

For the patients, who were alive at 30 days after TAVI (“survivors”), the two-year survival was 83%. Acute kidney injury (83% vs. 56%; log-rank *p* < 0.001) as well as catecholamine therapy (88% vs. 61%; log-rank *p* < 0.001) significantly influenced the two-year survival rate (Fig 1).

Kaplan–Meier estimates comparing outcomes between the two types of TAVI prostheses used did not show a significant difference in short- or long-term mortality rates.

### Postoperative data and Outcome

Transfemoral TAVI was technically successful in all patients, who were transferred to our ICU for post-interventional surveillance and care. The rates of catecholamine therapy upon admission to the ICU (93% vs. 29%; *p* < 0.001), stroke (20% vs. 1%; *p* < 0.001), cardiopulmonary resuscitation (60% vs. 6%; *p* < 0.001), and sepsis (27% vs. 2%; *p* < 0.001) were significantly higher in non-survivors compared with survivors (Table 2).

**Table 2:** Postoperative data.

	All patients n = 214	Survivor n = 199	Non-Survivor n = 15	p-value
ICU stay [days] mean±SD	5.1 ± 9.8	4.9 ± 9.9	8.0 ± 7.0	0.223
SAPS II, mean±SD	30.6 ± 6.6	29.9 ± 6.2	38.1 ± 7.0	<0.001
SAPS II Predicted Mortality, mean±SD	11.4 ± 9.1	10.5 ± 8.2	23.1 ± 11.7	<0.001
Catecholamines upon admission, n(%)	71 (33)	57 (29)	14 (93)	<0.001
Pacemaker Implantation, n(%)	30 (14)	28 (14)	2 (13)	0.856
Stroke, n(%)	4 (2)	1 (1)	3 (20)	<0.001
CPR, n(%)	20 (9)	11 (6)	9 (60)	<0.001
(Re)Intubation, n(%)	24 (11)	17 (9)	7 (47)	<0.001
AKI, n(%)	45 (21)	39 (20)	6 (40)	0.094
Bleeding, n(%)	23 (11)	19 (10)	4 (27)	0.056
Vascular Complications, n(%)	43 (20)	34 (17)	9 (60)	<0.001
RBCC, mean±SD	0.3 ± 0.5	0.3 ± 0.5	0.6 ± 0.5	0.007
Pneumonia, n(%)	11 (5)	8 (4)	3 (20)	0.012
Sepsis, n(%)	8 (4)	4 (2)	4 (27)	<0.001
30day mortality, n(%)	15 (7)	0 (0)	15 (100)	<0.001

**SAPS**: Simplified Acute Physiology Score, **ICU**: Intensive care unit, **CPR**: cardiopulmonary resuscitation, **AKI**: acute kidney injury, **RBCC**: red blood cell count

Vascular complications occurred in 20% of all patients (60% vs. 17%) and had an impact on both 30-day (21% vs. 4%; p < 0.001) and two-year mortality (35% vs. 19%; p = 0.012) rates.

### SAPS II

The mean SAPS II score was higher in the non-survivors than in the survivors (38.1 ± 7.0 vs. 29.9 ± 6.2; *p* < 0.001). Based upon these scores, the predicted mortality was 23.1 ± 11.7% and 10.5 ± 8.2% *p* < 0.001, respectively.

In contrast, the logistic EuroSCORE revealed no differences between the groups (22 ± 16% vs. 20 ± 12%; *p* = 0.555). Interestingly, both scores overestimated the actual mortality. Nevertheless, the SAPS II successfully discriminated between the groups, whereas the logistic EuroSCORE did not.

**Fig 2:**
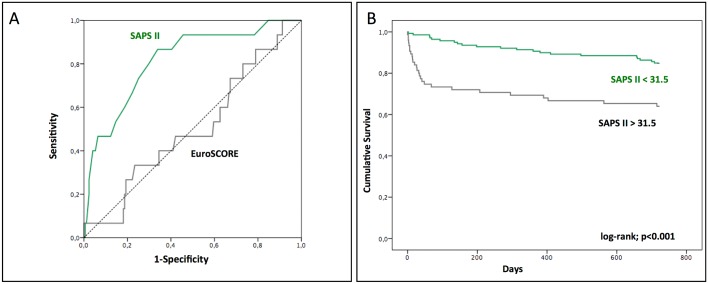
SAPS II. Receiver operating characteristic analysis for the accuracy of the EuroSCORE and SAPS II in predicting mortality among TAVI patients (A). Kaplan–Meier curve showing the survival of the study population for all consecutive patients undergoing TAVI, using a cut-off of 31.5 for SAPS II (B).

The SAPS II exhibited a superior discriminatory performance in comparison to the logistic EuroSCORE, with an AUROC of 0.82 (95% CI 0.70–0.93) and 0.51 (95% CI 0.36–0.66), respectively (Fig 2A). Using receiver operating characteristic curve analysis, a cut-off point of 31.5 proved to be a good predictor of mortality, with a sensitivity of 87% and a specificity of 76%. When this cut-off value was applied, the cumulative survival also showed clearly significant differences also at two years (85% vs. 64%; *p* < 0.001) (Fig 2B).

### Laboratory Values

In order to demonstrate the differences between both groups, four laboratory values were evaluated: creatinine, procalcitonin (PCT), brain natriuretic peptide, and troponin I. The maximum values of creatinine (2.46 ± 1.24 vs. 1.77 ± 1.16 mg/dL; *p* < 0.05), PCT (1.44 ± 1.84 vs. 0.51 ± 1.65 ng/mL; *p* < 0.05) and troponin I (4.46 ± 4.19 vs. 2.02 ± 2.25 ng/mL; *p* < 0.001) as well as BNP (996.89 ± 828.41 vs. 762.63 ± 963.65 pg/mL; p = 0.347) during the first 48 hours after ICU admission were significantly higher in the non-survivors compared with survivors.

**Fig 3:**
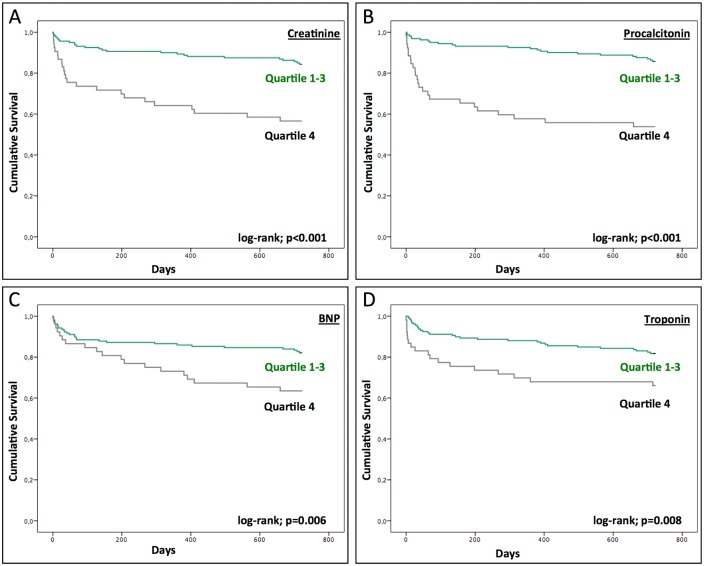
Laboratory Values. Kaplan–Meier curve for survival of all consecutive patients undergoing TAVI according to post-procedural levels of creatinine (A), procalcitonin (B), brain natriuretic peptide (C), and troponin (D), comparing the upper quartile [4] with the lower three quartiles [1–3].

In addition, our total cohort was divided into quartiles according to the maximum value of each biomarker in order to demonstrate the strength of the relationship between their increase and mortality. In this comparison, the upper quartile (with the highest values) had lower survival rates at two years (Fig 3), which was especially pronounced for creatinine (57% vs. 84%; *p* < 0.001) and PCT (54% vs. 86%; *p* < 0.001).

### Length of ICU stay

The mean ICU stay of our total cohort was 5.1 ± 9.8 days. The ICU stay of the non-survivors was in accordance with the postoperative course (see above), which was, on average, three days longer than that of the survivors (Table 2).

### Multivariate Analysis

In the multivariable model all tested variables showed no independent predictive value concerning two-year mortality.

## Discussion

This retrospective, single-centre study describes a transfemoral TAVI patient cohort admitted to a non-surgical ICU for post-interventional surveillance and analyses the risk assessment upon ICU admission including its implications for short- and mid-term outcomes.

Transfemoral TAVI has meanwhile become a well-established and standardized procedure with low complication rates. Yet, it appears that there is less focus on and standardization on of post-procedural care, and the post-procedural management seems to vary across centres. Nevertheless, post-procedural care is crucial due to the unique characteristics of TAVI patients, who are usually old, comorbid and frail. In this setting, risk assessment upon patient admission to the ICU appears of utmost importance for an improved patient management [21].

We found that almost all 30-day non-survivors died during their ICU stay. Although TAVI was successful in all patients, there were wide variations in the immediate post-procedural course of each patient varied widely. The rates of catecholamine therapy upon admission to the ICU, stroke, cardiopulmonary resuscitation, and sepsis were significantly higher in the non-survivors, and vascular complications negatively impacted survival.

Prognostic scores, such as SAPS II are widely used in the ICU setting. However, these scores have not been validated in patients with severe aortic stenosis, or in relation to TAVI procedures. In contrast to the EuroSCORE [22], which is widely used for preoperative risk assessment despite being known to not provide an accurate prediction of mortality in the setting of TAVI, the SAPS II is a good tool for estimating ICU mortality immediately after the TAVI procedure in this high-risk cohort and provides valuable information for other known predictors of mortality such as AKI, PCT, and myocardial injury. Yet, the SAPS II overestimated the rate of mortality in our study population. Although two specific TAVI risk scores have previously been presented [23,24] that improve the clinical decision-making process, both scores have not yet been applied to routine TAVI screening and also can only serve a moderate discriminatory function in the clinic. Therefore, the SAPS II might still be a valuable tool for the identification of patients who are at an increased risk during post-procedural monitoring. In our cohort, mortality predicted by SAPS II showed a distinct and significant difference between the two groups (23.1 ± 11.7% vs. 10.5 ± 8.2%; *p* < 0.001).

We examined various laboratory values as predictors of outcome in patients with TAVI. A post-interventional increase in troponin I after TAVI involving some type of myocardial injury is a known risk factor for a higher cardiac mortality, according to previous studies [25,26,27]. There are several possible reasons for myocardial injury during TAVI, such as episodes of extreme hypotension, the use of rapid pacing, or coronary embolism during balloon valvuloplasty and valve implantation [28].

A post-interventional increase in creatinine with the development of AKI has already been reported as an important risk factor for 30-day mortality (HR 4.9). Previous studies report the incidence of AKI after TAVI to be between 12% and 28%, which is comparable with the 21% found in our study [29–32]. Patients treated in the ICU after TAVI who are at risk for developing AKI should be observed and treated carefully, since AKI development is multifactorial and every attempt should be made to avoid this complication.

Developing systemic inflammatory response syndrome (SIRS) during cardiac surgery, particularly during cardiopulmonary bypass, is well known [33–35]. Furthermore, SIRS development has also been demonstrated after the TAVI procedures [7]. As PCT has been shown to be the most specific parameter related to the incidence of SIRS [7], we used PCT as a routine parameter for detection. Accordingly, a post-interventional increase in PCT was a strong predictor of 30-day and two-year mortality. Possible reasons for the development of SIRS include the aforementioned efforts to treat myocardial injury or the injury itself (e.g., balloon valvuloplasty, repositioning of prosthesis, rapid pacing) as well as the consequence of vascular complications and/or major bleeding events, as shown by previous studies [7]. Furthermore, the implications of SIRS coinciding with the development of AKI are discussed in several studies that examined inflammation parameters and AKI [7,36,37].

### Limitations

This is a single-centre, retrospective observational report with methodology-inherent potential bias that is common for these types of studies. Exemplary of such bias, the length of the ICU stay cannot be considered a valid retrospective endpoint due to the hesitation of clinicians to transfer patients to the regular ward in the initial years of our TAVI experience. Furthermore, patients that were monitored post-procedurally in one of the other five ICUs in our hospital were excluded from the analysis. However, the length of ICU stay and its reduction are the current focus of the consequences of implementing a minimalist, simplified TAVI procedure with excellent outcomes and a reduced use of health care resources. Use of the SAPS II might potentially help to better advise patients that require a prolonged ICU stay.

Although SAPS II is probably the most extensively used score for assessing illness severity in European ICU patients, various studies have reached dissimilar conclusions regarding its predictive value [13]. Furthermore, SAPS II relies on physiological parameters that are recorded within the first 48 h pre- and post-admission and does, thus, not take into account later deterioration.

## Conclusions

Post-operative care after TAVI is challenging due to procedural and postprocedural complications that may affect the outcome of the old, comorbid and frail patients. The identification of patients at higher risk of an unfavourable outcome upon admission to the ICU is therefore important in order to optimize the treatment with the inclusion of prevention strategies for complications such as AKI and SIRS. Hemodynamic management and the rapid recognition of vascular complications are among the main objectives of early postoperative ICU management requiring close postoperative monitoring and close fluid balance monitoring to prevent AKI.

The results of our study confirm that the SAPS II, obtained upon ICU admission, is applicable to TAVI patients and is useful for predicting 30-day mortality with an acceptable level of accuracy, while performing better than the EuroSCORE. Moreover, impaired renal function, the occurrence of AKI, an increase in PCT, and signs of myocardial injury are strong predictors not only of 30-day, but also two-year mortality after TAVI.

The SAPS II, in combination with biochemical markers, can be used for post-interventional risk stratification in the ICU to optimize patient management and provides valuable information in addition to other known predictors of mortality.

## Abbreviations

AKI: acute kidney injury
AUROC: area under the receiver operating characteristic curve
BNP: brain natriuretic peptide
EuroSCORE: European System for Cardiac Operative Risk Evaluation
ICU: intensive care unit
PCT: procalcitonin
SAPS: Simplified Acute Physiology Score
SIRS: systemic inflammatory response syndrome
TAVI: Transcatheter aortic valve implantation
VARC: Valve Academic Research Consortium
